# Copper-doped ZnO-ZrO_2_ solid solution catalysts for promoting methanol synthesis from CO_2_ hydrogenation

**DOI:** 10.1098/rsos.221213

**Published:** 2023-06-14

**Authors:** Fujiao Song, Wenqiang Cheng, Yang Yu, Yan Cao, Qi Xu

**Affiliations:** ^1^ School of Environmental Science and Engineering, Yancheng Institute of Technology, Yancheng 224051, People's Republic of China; ^2^ Department of Chemical and Biomolecular Engineering, National University of Singapore, Singapore

**Keywords:** copper-doped, ZnO-ZrO_2_, solid solution, CO_2_ hydrogenation

## Abstract

Copper-doped ZnO-ZrO_2_ solid solution catalysts were synthesized via co-precipitation for promoting CH_3_OH synthesis via hydrogenation of CO_2_. Various testing methods were applied to investigate the effect of various copper contents on the catalysts. The catalytic performance was evaluated by a fixed bed reactor. XRD, HRTEM and Raman spectra collectively indicated that a ZnO-ZrO_2_ solid solution catalyst with 3% Cu had a higher Cu dispersion, while the H_2_-TPR results confirmed that a catalyst with 3% Cu had more Cu active sites under low temperature H_2_ pretreatment. When the copper content increased to 5% and 10%, the catalyst showed a better Cu crystallinity and a worse Cu dispersion, which could have a negative effect. Therefore, the CO_2_ conversion and methanol yield with a 3% CuZnO-ZrO_2_ catalyst at 5 MPa, 250°C and 12 000 ml/(g h) increased by 8.6% and 7.6%, respectively. Moreover, the CH_3_OH selectivity and catalytic stability of the solid solution catalyst were better than those of the traditional CZA catalyst.

## 1. Introduction

With the progress and development of industrial society, increasing amounts of carbon dioxide have been released into the atmosphere [1,2]. CO_2_ is the main greenhouse gas, so it is necessary to reduce carbon dioxide emissions [3]. CO_2_ hydrogenation to methanol could provide a solution [4]. Methanol is one of the basic organic raw materials. It can be used to produce formic acid [5], olefins [6] and DME [7], and can also be used as a fuel product [8].

Among the reported catalysts, the Cu/ZnO/Al_2_O_3_ (CZA), Pd/Zn/CeO_2_ and Cu/Ni/CeO_2_ catalyst showed better performance. Pd/Zn/CeO_2_ is a noble metal catalyst and its large-scale application is limited by the higher cost, while the CZA catalysts with ultra-low cost have been extensively applied to CH_3_OH synthesis from hydrogenation [9]. Besides, CuO/ZnO/ZrO_2_ catalysts are also applied to methanol synthesis. Compared with alumina, zirconia has many excellent properties to provide copper dispersion sites and surface alkalinity, so the CuO/ZnO/ZrO_2_ catalysts could be more effective on the CO_2_ conversion to methanol [10]. In order to achieve better catalytic activity, co-precipitation [11], immersion method [12] and sol-gel [13] have been investigated extensively and coprecipitation is the most frequently used method.

Recently, Li [14] *et al*. developed a ZnO-ZrO_2_ solid solution with a CH_3_OH selectivity of 91% and CO_2_ conversion of 10%. The catalyst also showed excellent stability and provided new ideas. As we all know, the solid solution catalysts that have been reported at present are CeO_2_-based (e.g. ZrO_2_-CeO_2_, CuO-CeO_2_, MnO-CeO_2_) and ZrO_2_-CeO_2_ solid solution catalysts have excellent CO catalytic oxidation activity [15]. For Ce_x_Zr_1-x_ O_2_-based catalysts, noble metals usually act as active components [16], and meanwhile, transition metal oxides are widely used as surrogates [17]. Among these transition metal oxides, copper oxide modified Ce_x_Zr_1-x_O_2_ showed a higher catalytic ability of CO oxidation and NO removal. For the copper oxide modified Ce_x_Zr_1-x_O_2_ and CuO-CeO_2_ solid solution, the copper oxide species generally consist of three types and it has been widely accepted that highly dispersed CuO is more active than lattice doped and bulk phase CuO [18,19]. There are some limitations on catalytic CO_2_ conversion into methanol, while RWGS is the most likely secondary reaction. On the other hand, CO_2_ has strong chemical stability, so it is very important to study catalysts with fabulous catalytic activity and stability [20]. Apart from ZnO-ZrO_2_ solid solution, Li [21] *et al*. also reported a class of metal oxide solid solution catalysts: MaZrOx (Ma = Cd, Ga) for CO_2_ hydrogenation to methanol. Other solid solution catalysts, such as ZnO-ZrO_2_-MOx, were subsequently reported for the same application [22].

In this paper, when the Zn content was 20% (Zn/Zr = 1/4, molar ratio), ZnO and ZrO_2_ formed a solid solution structure. On this basis, several x% CuOZnO-ZrO_2_ were prepared by the co-precipitation (x% represents the molar percentage of copper). Generally, Cu^2+^ ions could embed into the lattice of CeO_2_ and form a solid solution at samll Cu/(Ce + Cu) proportions (less than 0.1). The ranges of copper doping content were less than 10% (*x* = 0, 0.5, 1, 3, 5, 10).

Cu-Zn binary catalysts have been widely used for CO_2_ hydrogenation to methanol, and there are many studies on introducing other supports or promoters into Cu-Zn catalysts. In our work, CuZnO-ZrO_2_ solid solution catalyst is the focus of research, and CZA is prepared as the comparison catalyst. Although there are a lot of catalysts that appear to be the same or similar in composition in literatures, the solid solution structure and catalytic performance of the CuZnO-ZrO_2_ catalyst in our work is significantly different from the other catalysts. After the ZnOZrO_2_ solid solution catalyst is doped with copper, a ternary solid solution is formed, which can improve its CO_2_ conversion while maintaining relatively high methanol selectivity. This is also the novelty of this study.

The representative physical and chemical properties of the catalysts were tested by various techniques. The facilitation of Cu components and the effects of Cu content on CO_2_ hydrogenation to CH_3_OH of the catalysts were studied and discussed.

## 2. Experimental section

### 2.1. Preparation of catalysts

Bimetallic oxide catalysts with various molar ratios of Zn-Zr, pure ZnO and ZrO_2_ were synthesized by a coprecipitation method, then the copper-doped ZnO-ZrO_2_ catalysts (the Cu/Zn/Zr molar ratio is 0–0.1/1/4) were also prepared by co-precipitation. The specific synthesis procedures are as follows: 1.49 g Zn(NO_3_)_2_·6H_2_O, 8.59 g Zr(NO_3_)_4_·5H_2_O and 0.18 g Cu(NO_3_)_2_·3H_2_O (taking the 3% CuZnO-ZrO_2_ catalyst as an example) were dissolved in distilled water, then 0.5 mol l^−1^ of Na_2_CO_3_ was dispersed in the aforementioned solution under stirring at 70°C to form a precipitate. The pH during precipitation was kept at 7.5 under continuous stirring for 1 h at 70°C, and then it was incubated for 3 h at 70°C. When cooled, the precipitate was centrifuged, washed with deionized water and dried at 105°C overnight. Finally, the product was calcined at 500°C in the air for 3 h. Other catalysts with different copper contents were prepared following the same method. The CZA catalysts (with molar ratios of Cu/Zn/Al = 6/3/1) were also synthesized by co-precipitation and their preparation process is consistent with the above statement. The amount of reagent is 7.2 g Cu(NO_3_)_2_·3H_2_O, 4.5 g Zn(NO_3_)_2_·6H_2_O, and 1.9 g Al(NO_3_)_3_·9H_2_O. For comparison, all reaction conditions, such as reaction temperature, reaction time and pH value, are completely consistent with the above experiments.

### 2.2. Characterization methods

The crystalline phase of the catalysts was investigated by a Panalytical X'Pert Pro X-ray diffractometer with Cu K*α* radiation (*λ* = 0.15416 nm), operating at 40 kv and 40 mA. N_2_ adsorption-desorption isotherms were obtained at −196°C by a Beckman Coulter SA 3100 type specific surface area and aperture measurement instrument (Beckman Coulter, Inc. Brea, USA) and the surface areas of the catalysts were calculated by the BET method. TG curves of the catalyst precursors was collected by an STA 449 TG-DSC synchronous thermal analyzer (50–750°C, in a steady air flow, heating rate = 5°C min^−1^). Raman spectra were recorded by a Raman microscope with a laser beam (*λ* = 325 nm). XPS spectra were used to measure the element composition and valence distribution of the catalysts (C1s peak at 284.8 eV).

H_2_-TPR was used to detect the reducibility. First, the catalyst in the tube was heated to 130°C in argon (50 ml min^−1^) keeping the samples at pretreatment for 2 h. Next, they were cooled to 50°C, then the samples were heated to 800°C in a 10% H_2_/Ar mixture and hydrogen consumption was measured by the AutoChemII2920 with a TCD detector. TEM images were observed on a TEM instrument at 200 kV accelerating voltage.

### 2.3. Catalytic activity measurements

CO_2_ hydrogenation to CH_3_OH was tested in a tubular reactor. The catalyst (0.5 g catalyst and 0.5 g quartz sand were mixed together and passed through a 40–80 mesh) was pretreated for 4 h in a 50% H_2_/N_2_ stream (20 ml min^−1^ and 0.2 MPa). The temperature was raised to 280°C, maintained for 4 h, and then cooled down. Next, the reaction was conducted at 1–5 MPa and 12 000 ml/(g h) GHSV, the reaction temperature was 190°C to 310°C and the volume ratio of H_2_ : CO_2_ : N_2_ was 72 : 24 : 4. The reaction products were collected after 3 h and then the reaction gas was analysed. Conversion of CO_2_, CH_3_OH selectivity and yield were calculated by the following equations (2.1)–(2.4).

2.1 $$X(\;CO_{2})=\left(f_{\mathrm{CO}}A_{\mathrm{CO}}+f_{{\mathrm{CH}}_{3}\mathrm{OH}}A_{{\mathrm{CH}}_{3}\mathrm{OH}}\right)/(f_{\mathrm{CO}}A_{\mathrm{CO}}+f_{{\mathrm{CH}}_{3}\mathrm{OH}}A_{{\mathrm{CH}}_{3}\mathrm{OH}}+f_{{\mathrm{CO}}_{2}}A_{{\mathrm{CO}}_{2}}),$$

2.2 $$S(\;CH_{3}\mathrm{OH})\;=(f_{{\mathrm{CH}}_{3}\mathrm{OH}}A_{{\mathrm{CH}}_{3}\mathrm{OH}})/\left(f_{{\mathrm{CH}}_{3}\mathrm{OH}}A_{{\mathrm{CH}}_{3}\mathrm{OH}}+f_{\mathrm{CO}}A_{\mathrm{CO}}\right),$$

2.3 $$S(\;\mathrm{CO})\;=f_{\mathrm{CO}}A_{\mathrm{CO}}/(f_{\mathrm{CO}}A_{\mathrm{CO}}+f_{{\mathrm{CH}}_{3}\mathrm{OH}}A_{{\mathrm{CH}}_{3}\mathrm{OH}}),$$

and

2.4 $$Y(\;CH_{3}\mathrm{OH})\;=X(\;CO_{2})\times S(\;CH_{3}\mathrm{OH})\;.$$

*A*_i_ means the peak area of the corresponding product and *f*_i_ means its correction factor.

## 3. Results and discussion

### 3.1. Catalysts characterization

#### 3.1.1. XRD analysis and Raman spectra analysis

**Figure 1.**
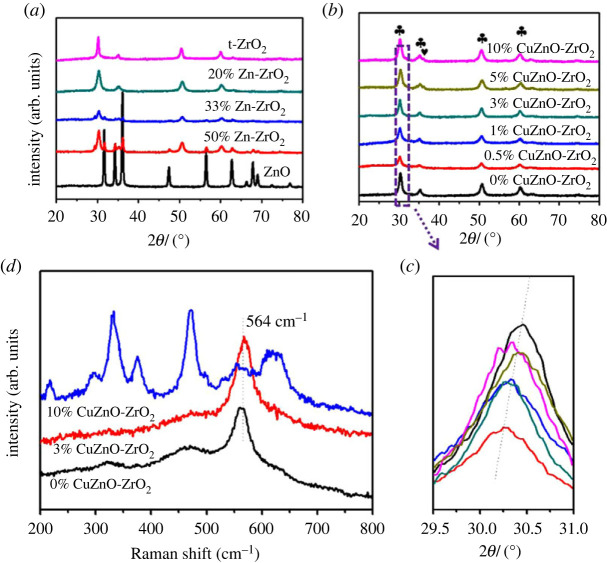
(*a*) XRD of ZnO-ZrO_2_ with various Zn content; (*b,c*) XRD of Cu-doped ZnO-ZrO_2_; (*d*) Raman spectra of the CuZnO-ZrO_2_.

Figure 1*a* exhibits the XRD of the ZnO-ZrO_2_ with various Zn contents. When the Zn molar content was 20%, the ZnO-ZrO_2_ formed a solid solution. Figure 1*b* shows the XRD of Cu-doped catalysts. Four sharp diffraction peaks are assigned to the crystal planes of t-ZrO_2_, respectively (JCPDS Card No. 50-1089) [23]. The diffraction peak of CuO could not be observed when the copper content was less than 5%, indicating the dispersity of Cu species is too high to detect by XRD. As the copper content increased, the peak of CuO was observed at 2*θ* of 36.1°(JCPDS Card No. 03-0879) in the 5% CuZnO-ZrO_2_ catalyst and in the 10% CuZnO-ZrO_2_ catalyst, which reveals that the Cu species existed in large particles of CuO [24]. Figure 1*c* shows the XRD of the t-ZrO_2_ (011) peak in all of the catalysts. It was found that the characteristic peak of ZrO_2_ (011) became broader, while the peak position offset to a smaller angle. This migration phenomenon may be due to the lattice distortion caused by copper ions entering the ZnO-ZrO_2_ tetragonal lattice. In table 1, the average crystalline sizes of the 0% CuZnO-ZrO_2_, 3% CuZnO-ZrO_2_ and 10% CuZnO-ZrO_2_ catalysts that were calculated by the Scherrer equation were 11.3 nm, 11.8 nm and 8.6 nm, respectively. The Raman spectra of the 0% CuZnO-ZrO_2_, 3% CuZnO-ZrO_2_ and 10% CuZnO-ZrO_2_ are shown in figure 1*d*. The peak of 0% CuZnO-ZrO_2_ at 564 cm^−1^ proves that there is a solid solution structure. Similarly, the peak at approximately 564 cm^−1^ was observed and CuO and Cu_2_O weren't observed, which proved that the Cu-Zn-Zr-O ternary solid solution was generated in the 3% CuZnO-ZrO_2_ catalyst. However, the peaks at 295, 331 and 625 cm^−1^ are assigned to the Raman-active Ag, 2Bg symmetry of CuO that existed in the 10% CuZnO-ZrO_2_ catalyst. The scanning results of the Raman spectra fit those of XRD.

**Table 1.** Textural properties data of the 0%CuZnO-ZrO_2_, 3%CuZnO-ZrO_2_ and 10%CuZnO-ZrO_2_ calculated from XRD, TGA and BET.

catalysts	crystallite size^a^ (nm)	total weight loss^b^ (%)	pore volume^c^ (cm^3^·g^−1^)	surface area^d^ (m^2^·g^−1^)
0%CuZnO-ZrO_2_	11.3	19.6	0.062	23.99
3%CuZnO-ZrO_2_	11.8	21.4	0.078	38.49
10%CuZnO-ZrO_2_	8.6	23.4	0.030	13.24

**^a^**Calculated from the Scherrer equation.

^b^Calculated by TG data.

^c^Calculated by the BJH model.

^d^Calculated by BET equation.

#### 3.1.2. TG analysis

**Figure 2.**
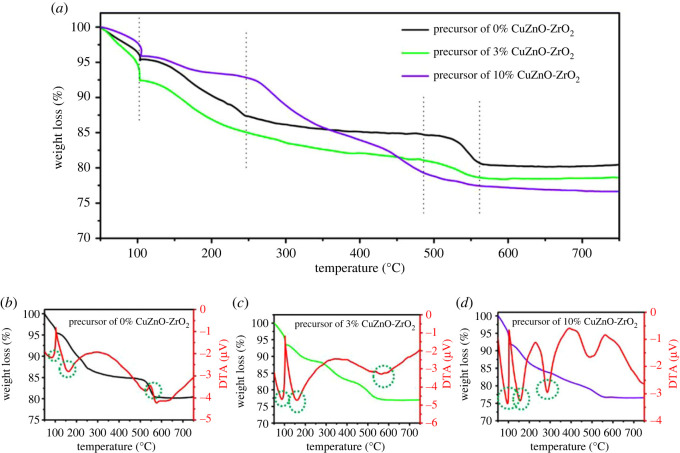
TG curves (*a*) for the precursor of the 0%CuZnO-ZrO_2,_ 3%CuZnO-ZrO_2_ and 10%CuZnO-ZrO_2_. TG-DTA curves of (*b*–*d*) for the precursor of the 0%CuZnO-ZrO_2_, 3%CuZnO-ZrO_2_ and 10%CuZnO-ZrO_2_, respectivity.

We investigated the thermal decomposition behaviour of the solid solution catalyst precursor with different copper doping amounts. The precursors of the 0% CuZnO-ZrO_2_, 3% CuZnO-ZrO_2_ and 10% CuZnO-ZrO_2_ are shown in figure 2*a*. The TG curve of the catalyst precursor consists of four stages: before 100°C, the weight loss was recognized as the loss of physically absorbed water, corresponding to the endothermic peaks in figure 2*b–d*. The decomposition of zinc carbonate 100–250°C corresponds to the endothermic peaks in figure 2*b–d*. Copper carbonate precipitates decompose at 250–450°C without an obvious endothermic peak in figure 2*c*, while significant endothermic peaks appear in figure 2*d*, which implies that there is copper in precursor of 10% CuZnO-ZrO_2_. A significant weight loss between 500 and 550°C reveals the formation of a solid solution structure in the precursors of the 0% CuZnO-ZrO_2_ and 3% CuZnO-ZrO_2_ catalysts, corresponding to the exothermic peak in figure 2*b*,*c*, while there was no obvious exothermic peak in the 10% CuZnO-ZrO_2_ precursor in figure 2*d* [25]. The total weight loss from the 0% CuZnO-ZrO_2_, 3% CuZnO-ZrO_2_ and 10% CuZnO-ZrO_2_ precursors were 19.6%, 21.4% and 23.4%, respectively (table 1). TG analysis showed that the 3% CuZnO-ZrO_2_ catalysts have a structure similar to the 0% CuZnO-ZrO_2_ catalyst, and proper copper doping is beneficial to form a solid solution structure, tallying with the XRD and Raman spectra analysis.

#### 3.1.3. BET analysis

**Figure 3.**
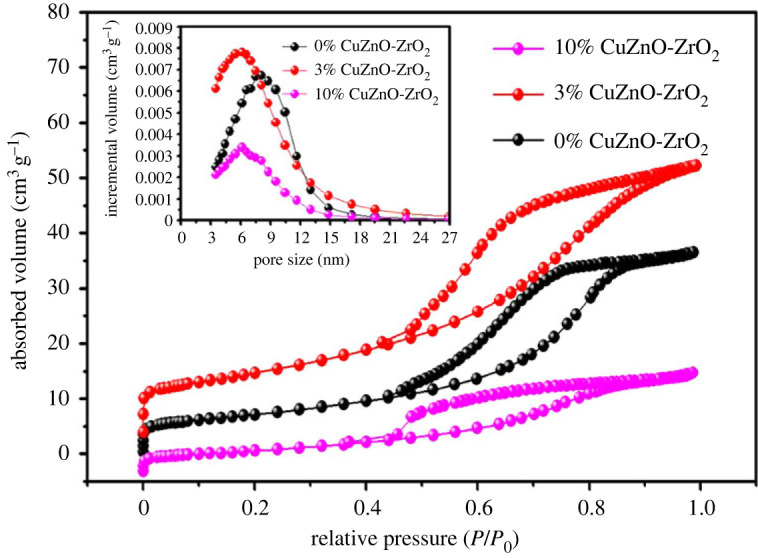
N_2_ adsorption-desorption isotherms and pore size distributions of the 0%CuZnO-ZrO_2_, 3%CuZnO-ZrO_2_ and 10%CuZnO-ZrO_2_.

Figure 3 shows the N_2_ adsorption–desorption isotherms of the 0% CuZnO-ZrO_2_, 3% CuZnO-ZrO_2_ and 10% CuZnO-ZrO_2_ catalysts. All of the isotherms belong to the classical type IV due to their mesoporous structure. Remarkably, a steep increase of adsorption at the P/P_0_ of 0.4–0.8 was observed due to capillary condensation of the adsorbate. The desorption isotherm is above the adsorption isotherms, and the hysteresis loop belongs to the H_2_-type [26]. The pore size distribution of the catalysts is shown in the inset of figure 3. Pores with a smaller radius can be detected by the DFT method, which is beyond the ability of the BJH methods to detect them [27]. It could be observed that peaks of the catalysts all appear at 3–15 nm, which is in the range of mesopore [28]. In table 1, the surface area of the 3% CuZnO-ZrO_2_ catalyst was 38.41 m^2^ g^−1^, higher than those of the 0% CuZnO-ZrO_2_ and 10% CuZnO-ZrO_2_ catalysts. Furthermore, the pore volume of the 3% CuZnO-ZrO_2_ catalyst was 0.078 cm^3^ g^−1^, which is also higher than those of the 0% CuZnO-ZrO_2_ and 10% CuZnO-ZrO_2_ catalysts. These results indicate that suitable copper doping in the ZnO-ZrO_2_ is critical to improve the catalysts' textural properties.

#### 3.1.4. TEM/HRTEM analysis

**Figure 4.**
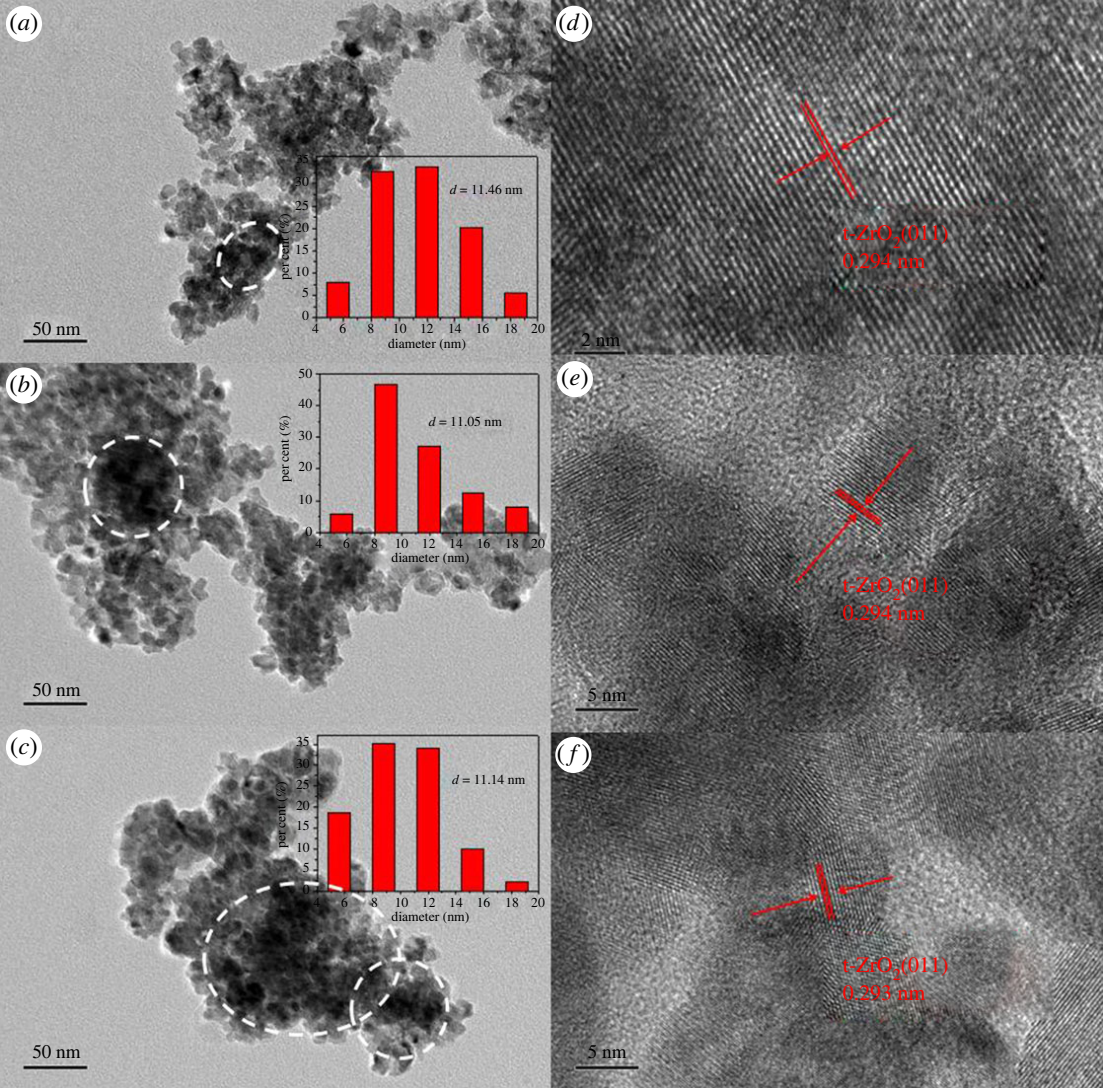
TEM images and size distribution: (*a*) 0%CuZnO-ZrO_2_, (*b*) 3%CuZnO-ZrO_2_ and (*c*) 10%CuZnO-ZrO_2_; HRTEM image of the catalyst: (*d*) 0%CuZnO-ZrO_2_, (*e*) 3%CuZnO-ZrO_2_ and (*f*) 10%CuZnO-ZrO_2_.

TEM morphology and size distribution of the catalysts are depicted in figure 4*a–c*. It can be observed that there are individual separated oxide nanoparticles in all of the catalysts. Sintering areas of three catalysts were marked by white circles, which increased with the copper content in the catalysts. Compared with the 0% CuZnO-ZrO_2_ and 3% CuZnO-ZrO_2_ catalysts, it is not easy to distinguish individual nanoparticles in the 10% CuZnO-ZrO_2_ catalyst, which indicates serious sintering. The particle size distribution showed that 66% of the granules were between 8 nm and 13 nm in 0% CuZnO-ZrO_2_, whose average particle size was 11.46 nm, while 74% granules are distributed in the same range in 3% CuZnO-ZrO_2_, whose average particle size was 11.05 nm, and 69% granules in 10% CuZnO-ZrO_2_ with an average particle size of 10.14 nm. When comparing with table 1, the average particle sizes of 0% CuZnO-ZrO_2_ and 3% CuZnO-ZrO_2_ were close to the crystalline size calculated by the Scherrer formula from the ZrO_2_ (011) peak, while the average size of the 10% CuZnO-ZrO_2_ catalyst was bigger than the crystalline size calculated by the Scherrer equation.

It can be concluded that the nanoparticles of the 3% CuZnO-ZrO_2_ catalyst are distributed uniformly. The HRTEM images of the 0% CuZnO-ZrO_2_, 3% CuZnO-ZrO_2_ and 10% CuZnO-ZrO_2_ catalysts are collected in figure 4*d–f*. The distance between two adjacent lattice stripes was approximately 0.294 nm, which is equal to the spacing of the (011) plane of t-ZrO_2_ [29]. The lattice fringes of CuO, Cu_2_O and ZnO were not found, which corresponds to the XRD results: no diffraction peaks of the ZnO and CuO or Cu_2_O can be observed in the patterns.

#### 3.1.5. XPS analysis

**Figure 5.**
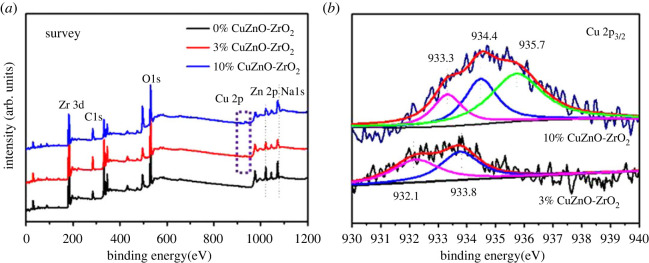
XPS patterns of the catalysts: (*a*) survey of 0%CuZnO-ZrO_2_, 3%CuZnO-ZrO_2_ and 10%CuZnO-ZrO_2_; (*b*) Cu 2p_3/2_ of 3%CuZnO-ZrO_2_ and 10%CuZnO-ZrO_2_.

Figure 5*a* displays XPS spectra of the 0% CuZnO-ZrO_2_, 3% CuZnO-ZrO_2_ and 10% CuZnO-ZrO_2_ catalysts, which confirm the coexistence of Zr, C, O, Cu, Zn and Na species. The copper content on the surface was higher than the experimental addition amount in 3% CuZnO-ZrO_2_, while the copper content of the surface was less than the experimental addition amount in 10% CuZnO-ZrO_2_. The Zn content was less than the theoretical value in the 0% CuZnO-ZrO_2_ and 3% CuZnO-ZrO_2_ catalysts, indicating that the ZnO surface was partly covered in CuO and ZrO_2_. Conversely, enriched Zn on the surface was observed in the 10% CuZnO-ZrO_2_ catalysts, and analogous results were reported for other Cu-Zn catalysts [30].

The XPS data are listed in table 2. The binding energy of Zn 2p3/2 at 1021 eV and Zr 3d_5/2_ at 182 eV can be assigned to ZnO and ZrO_2_, respectively [31]. The Cu 2p_3/2_ XPS spectra are exhibited in figure 5*b*. The binding energy of 10% CuZnO-ZrO_2_ is greater than 3% CuZnO-ZrO_2_, and the peak at 932.1 eV corresponds to Cu^0^ or Cu^+^, which indicates a strong effect between Cu and the solid solution. The peak at 933.8 eV of 3% CuZnO-ZrO_2_ and the peak at 933.4 eV and 935.7 eV of 10% CuZnO-ZrO_2_ correspond to Cu^2+^ [32].

**Table 2.** XPS data of the Cu-doped solid solution catalysts. Value in parentheses was theoretical concentration by normalized to the total metal content.

catalysts	surface atomic content of metals (%)			Zn/(Zn + Zr) molar ratio (%)	binding energy(eV)		
	Cu	Zn	Zr		Cu 2p_3/2_	Zn 2p_3/2_	Zr 3d_5/2_
0%CuZnO-ZrO_2_	—	16.5 (20)	83.5 (80)	16.5 (20)	—	1021.7	182.0
3%CuZnO-ZrO_2_	3.19 (3)	11.6 (19.4)	85.2 (77.6)	12.0 (20)	933.3	1021.5	181.9
10%CuZnO-ZrO_2_	7.05 (10)	22.3 (18)	70.6 (72)	24.25 (20)	934.4	1021.2	181.8

#### 3.1.6. H_2_-TPR analysis

**Figure 6.**
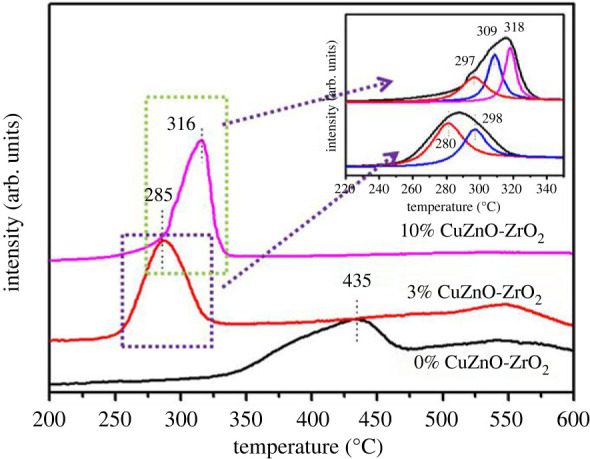
H_2_-TPR patterns of 0%CuZnO-ZrO_2_, 3%CuZnO-ZrO_2_ and 10%CuZnO-ZrO_2_.

H_2_-TPR profiles were shown in figure 6. Obviously, the reduction temperatures were much lower for the 3% CuZnO-ZrO_2_ and 10% CuZnO-ZrO_2_ catalysts than for the 0% CuZnO-ZrO_2_ catalyst, which indicates the solid solutions containing copper are more easily reduced than a copper free system. Both the 3% CuZnO-ZrO_2_ and 10% CuZnO-ZrO_2_ catalysts exhibited a broad reduction peak at 250–350°C as shown in the inset of figure 6; the temperature is higher than for traditional Cu-based catalysts [33]. In the document of CuO-CeO_2_, the first peak, the second peak and the third peak are designated as *α*, *β* and *γ* peak, respectively, where the *α* peak represents the CuO interacting with CeO_2_, the *β* peak originates from the deoxidation of highly dispersed CuO granules, and the *γ* peak is related to the deoxidation of bulk CuO [34]. Compared with the 10% CuZnO-ZrO_2_ catalyst, the *α* and *β* peaks of the 3% CuZnO-ZrO_2_ catalyst both moved to a lower temperature and the *γ* peak only existed in 10% CuZnO-ZrO_2_, which corresponds to the results of XRD, Raman and other characterizations.

### 3.2. Evaluation of the catalytic activity

#### 3.2.1. Effect of copper content

**Figure 7.**
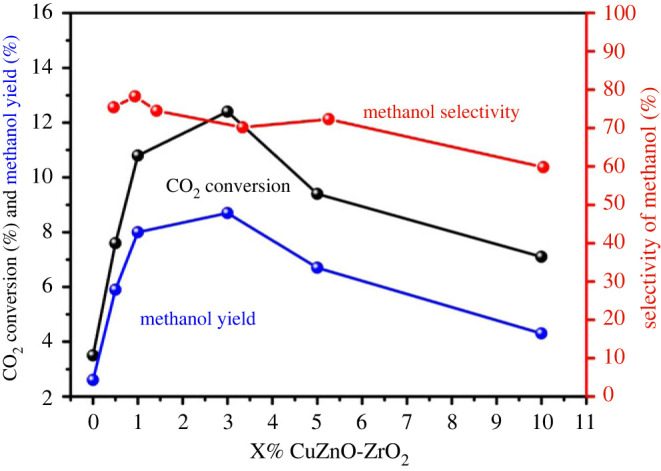
Effect of copper content on CO_2_ hydrogenation performance of the Cu-doped ZnO-ZrO_2_ solid solution.

The performance of CO_2_ hydrogenation on the CuZnO-ZrO_2_ solid solution is shown in figure 7. With the increase of Cu percentage in the solid solution, an obvious rise in CO_2_ conversion and CH_3_OH yield occurred when the copper addition amount was lower than 3%, while a decrease appeared when the copper amount was between 3% and 10%. However, the methanol selectivity did not change sharply with the copper content. A maximum catalytic activity was observed at a copper content of 3% in the solid solution catalyst. It could be concluded that the CO_2_ conversion increased significantly when copper was doped into the solid solution catalyst, and the optimal addition amount was 3%.

#### 3.2.2. Effect of reaction temperature

**Figure 8.**
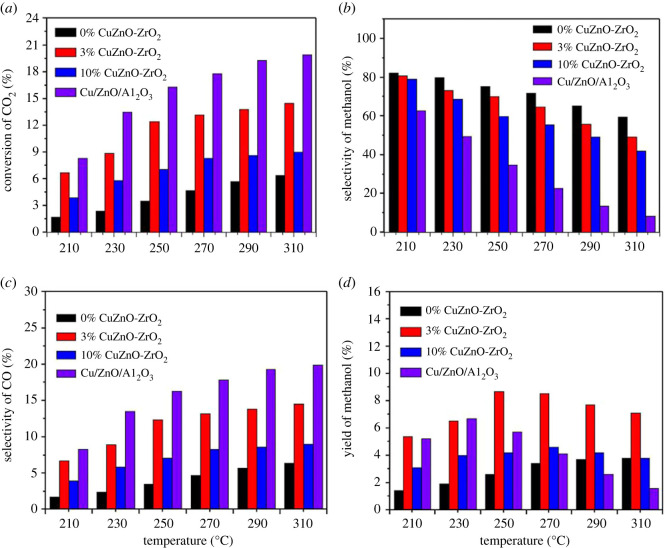
(*a*) Effect of the reaction pressure on CO_2_ conversion, (*b*) CH_3_OH selectivity, (*c*) CO selectivity, (*d*) CH_3_OH yield. Reaction condition: *P* = 2MPa, H_2_/CO_2_ = 3/1, GHSV = 12 000 ml/(g h).

The catalytic performance of the temperature dependence (210–310°C) is shown in figure 8. As is apparent in figure 8*a–c*, CO_2_ conversion increased with temperature, attended by a decline of CH_3_OH selectivity and an increase of CO selectivity, which is due to the competition between CH_3_OH synthesis and RWGS. The CH_3_OH synthesis is an exothermic reaction, but RWGS is the opposite. As seen in figure 8*a*, CO_2_ conversion of the traditional CZA catalyst was greater than the copper-doped ZnO-ZrO_2_ solid solution, while the CH_3_OH selectivity was the opposite (figure 8*b*). Compared with the solid solution catalysts, the CH_3_OH selectivity of the CZA catalyst decreased significantly with temperature. The solid solution catalyst in a high temperature reaction zone still maintains high methanol selectivity, which provides a theoretical basis C–C coupling reactions on the modified catalyst [23]. As shown in figure 8*d*, the CH_3_OH yield has a maximum value for all of the catalysts. However, the maximum CH_3_OH yield for each of the catalysts corresponds to a different critical temperature, which is 250°C for 3% CuZnO-ZrO_2_, 270°C for 10% CuZnO-ZrO_2_, and 230°C for CZA catalyst. Clearly, the maximum CH_3_OH yield reveals the critical point of the transformation from dynamics to thermodynamics [35]. When the methanol yield reaches the maximum value, the reaction temperature of the solid solution is greater than the CZA catalyst, which also indirectly indicates that the solid solution catalyst is not easily deactivated at high temperatures.

#### 3.2.3. Effect of reaction pressure

**Figure 9.**
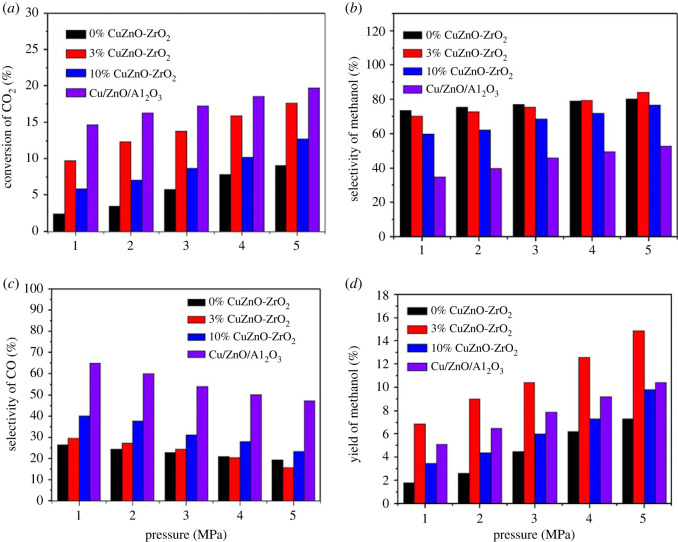
(*a*) Effect of the reaction pressure on CO_2_ conversion, (*b*) CH_3_OH selectivity, (*c*) CO selectivity, (*d*) CH_3_OH yield, Reaction condition: *T* = 250°C, H_2_/CO_2_ = 3/1, GHSV = 12 000 ml/(g h).

CO_2_ hydrogenation on ZnO-ZrO_2_ solid solution catalysts with various copper contents and CZA catalysts was studied in the reaction pressure range of 1–5 Mpa, as shown in figure 9. CO_2_ conversion, CH_3_OH selectivity and yield increased with reaction pressure (figure 9*a*,*b,d*), attended by a decline of CO selectivity (figure 9*c*). The number of molecules is reduced in CO_2_ hydrogenation to CH_3_OH, so the catalytic reaction will move into a positive direction with an increasing reaction pressure, and catalytic activity will increase significantly in the meantime. At 5 MPa, the CO_2_ conversion is 17.7%, the methanol selectivity is 84.1%, and then the highest methanol yield of 14.9% was obtained using 3% CuZnO-ZrO_2_. Compared with the ZnO-ZrO_2_ solid solution without copper addition, the CO_2_ conversion and CH_3_OH yield were increased by 8.6% and 7.6%, respectively. The highest CO_2_ conversion of 19.7% was found with the CZA catalyst, while the methanol selectivity was relatively low, and thus the methanol yield was not high.

#### 3.2.4. Stability analysis

**Figure 10.**
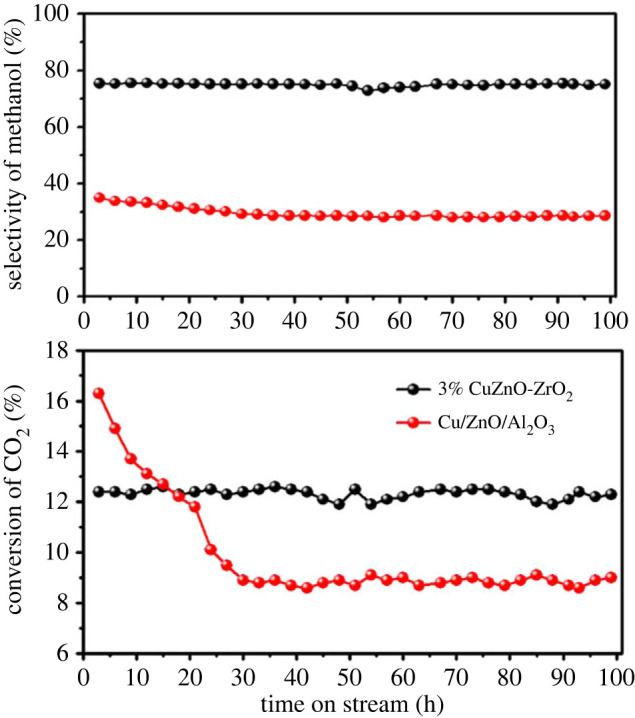
The catalytic activity with reaction time over the catalysts. Reaction conditions: *T* = 250°C, *P* = 2MPa, GHSV = 12 000 ml/(g h), H_2_/CO_2_ = 3/1.

The stability of the catalyst is also a key performance in CH_3_OH synthesis. The effect of the reaction time on the catalytic stability was investigated for 100 h. In figure 10, the CO_2_ conversion of CZA catalyst decreased from 16.27% to 8.92% with an attenuation rate of 45.2%, while the methanol selectivity decreased from 36.1% to 29.2% with an attenuation rate of 19.1%. The marked deactivation phenomenon of the CZA catalyst can be attributed to sintering of Cu particles. Howcver, the 3% CuZnO-ZrO_2_ catalyst had the highest stability for CO_2_ methanation to CH_3_OH and could run for 100 h almost without any inactivation.

#### 3.2.5. Catalytic performance comparisons

Although there are a lot of catalysts that appear to be the same or similar in composition in literature, the solid solution structure and catalytic performance of the CuZnO-ZrO_2_ catalyst in our work is significantly different from the other catalysts. Here, the catalysts closely related to this paper, such as CuZnZr, CuZnAl catalysts prepared by different methods and other solid solution catalysts, were listed in table 3 for catalytic performance comparisons with our work.

**Table 3.** Catalytic performance comparisons of catalysts in this work and in literature. G, gas-hourly space velocity (h^−1^); W, weight-hourly space velocity (ml gcat^−1^ h^−1^).

catalyst	synthetic method/novelty	H_2_/CO_2_ ratio	*T* (°C)	*P* (MPa)	space velocity	XCO_2_ (%)	SCH_3_OH (%)	Ref.
CuZnZr	co-precipitation/solid solution structure	3	250	2	12 000G	12.4	70.2	This work
CuZnZr	co-precipitation/solid solution structure	3	250	5	12 000G	19.7	81.4	This work
ZnZr	co-precipitation/solid solution structure	3	320	5	24 000G	10	86	[14]
Cd/GaZr	co-precipitation/solid solution structure	3	—	5	24 000G	12.4	80	[21]
MgZnZr	co-precipitation/solid solution structure	3	320	3	2000G	12.9	81.5	[22]
CuZnZr	solvothermal preparation	3	240	3	2400w	15.7	45	[36]
CuZnZr	surfactant-assisted coprecipitation	3	240	3	3600G	12.1	54.1	[37]
CuZnZr	combustion synthesis	3	240	3	3600G	17	56.2	[38]
CuZnZr	glycine–nitrate combustion synthesis	3	220	3	3600G	12.0	71.1	[39]
CuZnZr	ultrasonic impulse co-precipitation	3	350	3	3100G	18.7	53.6	[40]
CuZnAl	co-precipitation	3	250	2	12 000G	16.27	36.1	This work
CuZnAl	oxalate gel co-precipitation	3	240	2	3600G	20	31	[41]
CuZnAl	-	3	206	2	6000G	11	75	[42]
CuZnAl	oxalate gel co-precipitation	3	170	5	—	25	73	[43]
CuZnAl	deposition precipitation	3	300	5	2000w	25	26	[44]
CuZnAl	deposition precipitation	3	180	4.5	—	—	59	[45]
CuZnAl	a commercial catalyst for a benchmark study	5/3-3	210–250	3–5	500–1000w	—	28–54	[46]

As shown in table 3, the CO_2_ conversion and CH_3_OH selectivity of the reported solid solution catalysts ZnZr, Cd/GaZr and MgZnZr are 10–12.9% and 80–86% respectively at 3–5 MPa, 320°C and H_2_: CO_2_ molar ratio of =3 : 1 [14,21,22]. The CO_2_ conversion of CuZnZr catalysts prepared by different methods under different conditions varies from 12.0% to 18.7%, and the CH_3_OH selectivity varies from 45% to 71.1% [36–40]. The CO_2_ conversion of CuZnAl catalysts prepared by different methods under different conditions varies from 11% to 25%, and the CH_3_OH selectivity varies from 26% to 75% [41–45]. In a benchmark study of the catalytic performance of CuZnAl catalysts, the CH_3_OH selectivity varies from 28% to 54% at the pressure range of 3–5 MPa, temperature range of 210–250°C, H_2_: CO_2_ molar ratio range of 5 : 3–3 : 1 and flow rate range of 500–1000 NmL g_cat_^−1^ min^−1^ [46].

In our study, the CO_2_ conversion and CH_3_OH selectivity of CZA catalyst are 16.27% and 36.1% respectively at 2 MPa, 250°C, 12 000 ml/(g h) and H_2_: CO_2_ molar ratio of = 3 : 1, which is roughly in line with the range reported in the above literature. The CO_2_ conversion and CH_3_OH selectivity of CuZnO-ZrO_2_ solid solution catalyst is 12.4–19.7% and 70.2–81.4% respectively at 2–5 MPa, 250°C, 12 000 ml/(g h) and H_2_: CO_2_ molar ratio of = 3 : 1, whose catalytic activity level appears to be above average. Due to the different reaction conditions used in various studies, the comparison of catalytic performance has some reference value but cannot be completely trusted.

## 4. Conclusion

ZnO-ZrO_2_ solid solution catalysts with various copper contents were synthesized for CO_2_ hydrogenation to CH_3_OH. The XRD, HRTEM and Raman spectra analysis results showed that there is a solid solution structure in the 3% CuZnO-ZrO_2_ catalyst, and its specific surface area is 38.408 m^2^ g^−1^, higher than those of the 0% CuZnO-ZrO_2_ and 10% CuZnO-ZrO_2_ catalysts. The H_2_-TPR analysis proved that the 3% CuZnO-ZrO_2_ catalyst exhibited higher catalytic performance at a relatively lower reaction temperature. As against 10% CuZnO-ZrO_2_, 3% CuZnO-ZrO_2_ contains more dispersed copper species, whose catalytic activity is higher than that of the bulk CuO species. The XPS analysis indicates a strong interaction between Cu and the ZnO-ZrO_2_ solid solution. The CO_2_ conversion and CH_3_OH yield of the 3% CuZnO-ZrO_2_ reached 17.7% and 14.9%, respectively (5 MPa, 250°C and 12 000 ml/(g h)), while those of the 0% CuZnO-ZrO_2_ were 9.1% and 7.3%, respectively. Compared with the traditional CZA catalyst, the Cu-doped solid solution had higher methanol selectivity and better high temperature resistance.

## Data Availability

The data have been uploaded as electronic supplementary material [47].
